# Is neoadjuvant chemotherapy necessary for T2N0-1M0 hormone receptor-positive/HER2-negative breast cancer patients undergoing breast-conserving surgery?

**DOI:** 10.1007/s00432-024-05810-6

**Published:** 2024-05-30

**Authors:** Dandan Liu, Lidan Chang, Qian Hao, Xueting Ren, Peinan Liu, Xingyu Liu, Yumeng Wei, Meng Wang, Hao Wu, Huafeng Kang, Shuai Lin

**Affiliations:** 1 The Comprehensive Breast Care Center, The Second Affiliated Hospital of Xi’an Jiaotong University, Xi’an, 710061 Shaanxi China; 2 School of Basic Medical Sciences, Xi’an Key Laboratory of Immune Related Diseases, Xi’an Jiaotong University, Xi’an, Shaanxi China

**Keywords:** Neoadjuvant chemotherapy, HR-positive/HER2-negative, Breast cancer, SEER

## Abstract

**Introduction:**

For HR-positive/HER2-negative patients who can undergo breast-conserving surgery (BCS) but have a tumor size of 2–5 cm or 1–3 lymph node metastases, neoadjuvant chemotherapy (NAC) is still controversial.

**Methods:**

Patients with T2N0-1M0 HR-positive/HER2-negative BC who underwent BCS between 2010 and 2017 were selected from the SEER database. Propensity score matching (PSM) was used to minimize the influence of confounding factors. The overall survival (OS) and breast cancer-specific survival (BCSS) of patients were estimated by Kaplan‒Meier curves and Cox proportional hazard models. Independent prognostic factors were included to construct a nomogram prediction model.

**Results:**

A total of 6475 BC patients were enrolled, of whom 553 received NAC and 5922 received adjuvant chemotherapy (AC). In the T2N0-1M0 population and T2N1M0 subgroup, AC patients before PSM had better OS and BCSS than NAC patients. After PSM, there was no significant difference in OS or BCSS between the two groups. However, in the T2N0M0 subgroup, there was no difference in survival between the AC and NAC groups before and after PSM. Stratified analysis revealed that for complete response (CR) patients, survival was roughly equivalent between the NAC and AC groups. However, the survival of no response (NR) and partial response (PR) patients was significantly worse than that of AC patients. Cox analysis revealed that radiotherapy after BCS was an independent protective factor for OS. NAC is an independent risk factor for NR and PR patients. The nomogram has good prediction efficiency.

**Conclusion:**

NAC before BCS is not necessary for T2N0-1M0 HR-positive/HER2-negative BC patients.

**Supplementary Information:**

The online version contains supplementary material available at 10.1007/s00432-024-05810-6.

## Introduction

According to recent cancer statistics, breast cancer (BC) is still the most common cancer and the second most common cause of cancer-related mortality in women worldwide(Siegel et al. 2024). The incidence of BC in women continues to increase annually(Giaquinto et al. 2022), so more precise individual treatment is becoming essential. BC is classified into hormone receptor (HR)-positive/HER2-negative, HER2-positive, and triple-negative tumor subtypes based on estrogen or progesterone receptor expression and HER2 gene amplification (Burstein et al. 2021; Waks and Winer 2019). HR-positive/HER2-negative BC is the most common subtype of BC (Giaquinto et al. 2022; Waks and Winer 2019). The molecular subtype of BC is the key to guiding the formulation of the best individualized treatment plan (Goldhirsch et al. 2013; Waks and Winer 2019).

Although most patients with early-stage BC receive surgery combined with postoperative adjuvant therapy, the use of neoadjuvant chemotherapy (NAC) is increasing (Murphy et al. 2018). NAC refers to adjuvant chemotherapy prior to surgery and was initially reserved for the treatment of inoperable advanced patients (Rubens et al. 1980). However, with advancements in research, NAC indications have been extended to operable BC (Bear et al. 2003). It can not only reduce the total resection rate and improve the breast-conserving surgery (BCS) rate (Golshan et al. 2015, 2016; Kim et al. 2019) but also reduce the scope of axillary surgery(King and Morrow 2015; Mamtani et al. 2016; Morrow and Khan 2020), which plays an important role in the treatment of BC. In particular, the treatment response is significant in HER2-positive and triple-negative breast cancer (TNBC) patients (Cortazar et al. 2014; Murphy et al. 2018). The guidelines recommend neoadjuvant systemic therapy for patients with HER2-positive BC or TNBC (Korde et al. 2021). However, for patients with HR-positive/HER2-negative tumors, NAC is mainly used to shrink the primary tumor for smaller breast surgery (Torrisi et al. 2021). The pathological complete response (pCR) rate is an important indicator of the efficacy of NAC. HER2-positive BC and TNBC can achieve pCR rates of up to 45% (Boughey et al. 2014; Rouzier et al. 2005), but patients with HR-positive/HER2-negative tumors have PCR rates of only 0–18% after NAC (Torrisi et al. 2021), which is much lower than those of patients with the other two subtypes. However, pCR was strongly associated with survival in patients with HER2-positive BC and TNBC but not in HR-positive/HER2-negative BC patients (von Minckwitz et al. 2012). Despite the low pCR rate, HR-positive/HER2-negative BC patients generally have an improved long-term prognosis (Torrisi et al. 2021) and good locoregional recurrence-free survival (Caudle et al. 2012). Moreover, some studies have reported that there is no significant difference in the BCS conversion rate and tumor response rate between HR-positive/HER2-negative patients and patients with other types of BC (Kim et al. 2019). Thus, the use of NAC in the HR-positive/HER2-negative population showed an increasing trend (Murphy et al. 2018), even if the pCR rates were not comparable to those in the other two subtypes of breast cancer.

NAC is mainly used for BC patients whose tumor size is > 5 cm, who have axillary lymph node metastasis, who are HER2-positive BC, or who are TNBC (Jiang et al. 2022). However, it is controversial whether to perform NAC in HR-positive/HER2-negative BC patients who are eligible for BCS but with a tumor size of 2–5 cm or 1–3 lymph node metastases (Jiang et al. 2022; Spring et al. 2022). Therefore, our study focused on T2N0-1M0 HR-positive/HER2-negative BC patients who could undergo BCS, aiming to explore whether NAC could improve survival in this patient population where BCS is feasible.

## Materials and methods

### Selection of study subjects

**Fig. 1:**
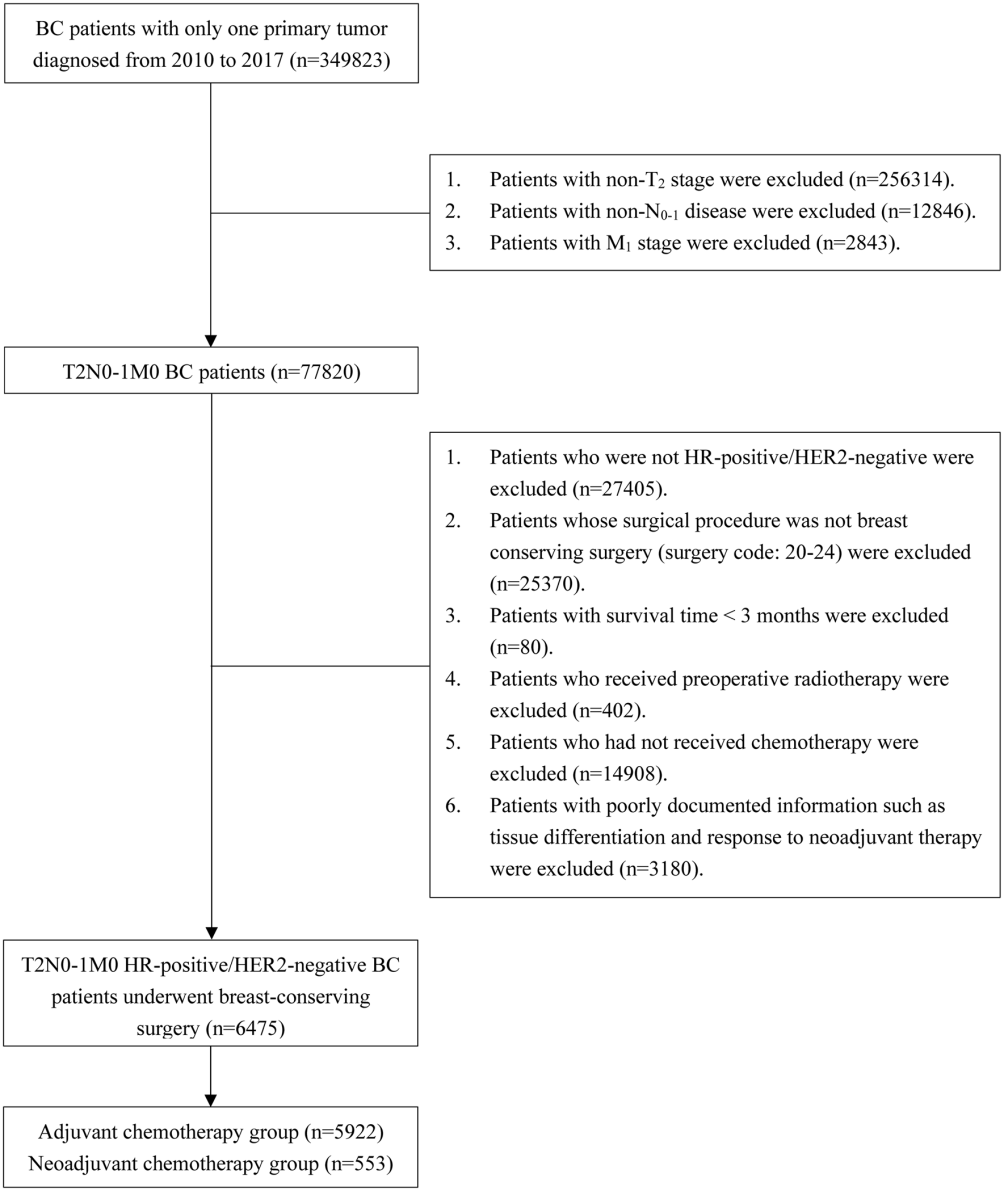
Flowchart of patients. There were 6475 patients who met the inclusion and exclusion criteria. A total of 553 patients received neoadjuvant chemotherapy, whereas 5922 patients received adjuvant chemotherapy

Patients who were diagnosed with T2N0-1M0 HR-positive/HER2-negative BC and treated with BCS were enrolled from the Surveillance, Epidemiology, and End Results (SEER) database (17regs, 2022nov sub). The inclusion criteria for patients were as follows: (1) had T2N0-1M0 BC diagnosed by pathology between 2010 and 2017 and (2) had a single primary tumor. The exclusion criteria were as follows: (1) had a BC subtype other than HR-positive/HER2-negative; (2) underwent surgery other than BCS; (3) had a survival time < 3 months; (4) received preoperative radiotherapy; (5) did not receive chemotherapy; (6) had uncertain or unknown information, such as the degree of tissue differentiation. The specific screening procedure is shown in Fig. 1. The extracted clinicopathological data included year of diagnosis, age, race, marital status, histological type, differentiation degree, T-N-M stage, BC subtype, surgery, radiotherapy, chemotherapy, neoadjuvant treatment response, and survival data.

### Ethical information

The SEER database has an open-access policy, and all personal information has been de-identified. Therefore, no additional ethical approval or informed consent was required.

### Statistical analysis

Patients were divided into two groups according to whether they had received NAC. Descriptive statistics were used for baseline characteristics, and the Pearson χ2 test was used to test the balance between groups. To improve the comparability between groups, 1:1 propensity score matching (PSM, method = "nearest", caliper = 0.02) was used to reduce the influence of confounding factors. The endpoints analysed were overall survival (OS) and breast cancer-specific survival (BCSS). Kaplan–Meier curves and log-rank tests were used to compare the effect of NAC on survival. Cox proportional hazards models were used to evaluate the independent prognostic factors of patients. The adjusted hazard ratio (AHR) was calculated based on multivariate Cox regression analysis. Independent prognostic factors identified by Cox analysis were included in the construction of the nomogram, and the c-index and calibration curves were used to evaluate the predictive performance of the nomogram. Two-sided P < 0.05 was considered to indicate a statistically significant difference in this study. All analyses and plots were performed using R software (4.2.3) and related R packages.

## Results

### Baseline characteristics of T2N0-1M0 HR-positive/HER2-negative BC patients

A total of 6475 patients with T2N0-1M0 HR-positive/HER2-negative BC who underwent BCS were included in this study. Of these patients, 553 received NAC, and 5922 received adjuvant chemotherapy (AC). Most of the patients were > 50 years old (65.9%), white (76.9%), married (61.9%), ductal (85.2%), poorly or undifferentiated (47.9%) and treated with radiotherapy (84.9%). The NAC and AC groups differed in terms of age, race, marital status, histological type, and degree of differentiation. Among the patients receiving NAC, the proportion of patients under 50 years old and patients with poor differentiation increased. After PSM to balance confounding factors, the χ2 test suggested balance and comparability between the two groups (Table 1).

**Table 1:** Baseline demographic characteristics of T2N0-1M0 HR-positive/HER2-negative breast cancer patients (n = 6475)

variable	T2N0-1M0 (n = 6475)					
	Before PSM			After PSM		
	AC N = 5922 (%)	NAC N = 553 (%)	P	AC N = 553 (%)	NAC N = 553 (%)	P
Age			< 0.001			1.000
< = 50	1960 (33.1)	250 (45.2)		250 (45.2)	250 (45.2)	
> 50	3962 (66.9)	303 (54.8)		303 (54.8)	303 (54.8)	
Race			0.019			0.985
White	4579 (77.3)	399 (72.2)		401 (72.5)	399 (72.2)	
Black	661 (11.2)	79 (14.3)		77 (13.9)	79 (14.3)	
Other^a^	682 (11.5)	75 (13.6)		75 (13.6)	75 (13.6)	
Marital status			0.029			0.952
Married	3690 (62.3)	318 (57.5)		316 (57.1)	318 (57.5)	
Not-married^b^	2232 (37.7)	235 (42.5)		237 (42.9)	235 (42.5)	
Histologic type			< 0.001			0.988
Ductal	5011 (84.6)	506 (91.5)		507 (91.7)	506 (91.5)	
Ductal and lobular	426 ( 7.2)	23 ( 4.2)		22 ( 4.0)	23 ( 4.2)	
Lobular	485 ( 8.2)	24 ( 4.3)		24 ( 4.3)	24 ( 4.3)	
Grade			0.005			0.998
Grade I	494 ( 8.3)	42 ( 7.6)		42 ( 7.6)	42 ( 7.6)	
Grade II	2628 (44.4)	210 (38.0)		211 (38.2)	210 (38.0)	
Grade III or IV	2800 (47.3)	301 (54.4)		300 (54.2)	301 (54.4)	
Radiation			0.314			0.930
No	904 (15.3)	75 (13.6)		77 (13.9)	75 (13.6)	
Yes	5018 (84.7)	478 (86.4)		476 (86.1)	478 (86.4)	

^a^Other American Indian/AK Native, Asian/Pacific Islander

^b^Not-married single (never married), separated, divorced, widowed, unmarried or domestic partner

### Survival analysis of the overall study subjects

**Fig. 2:**
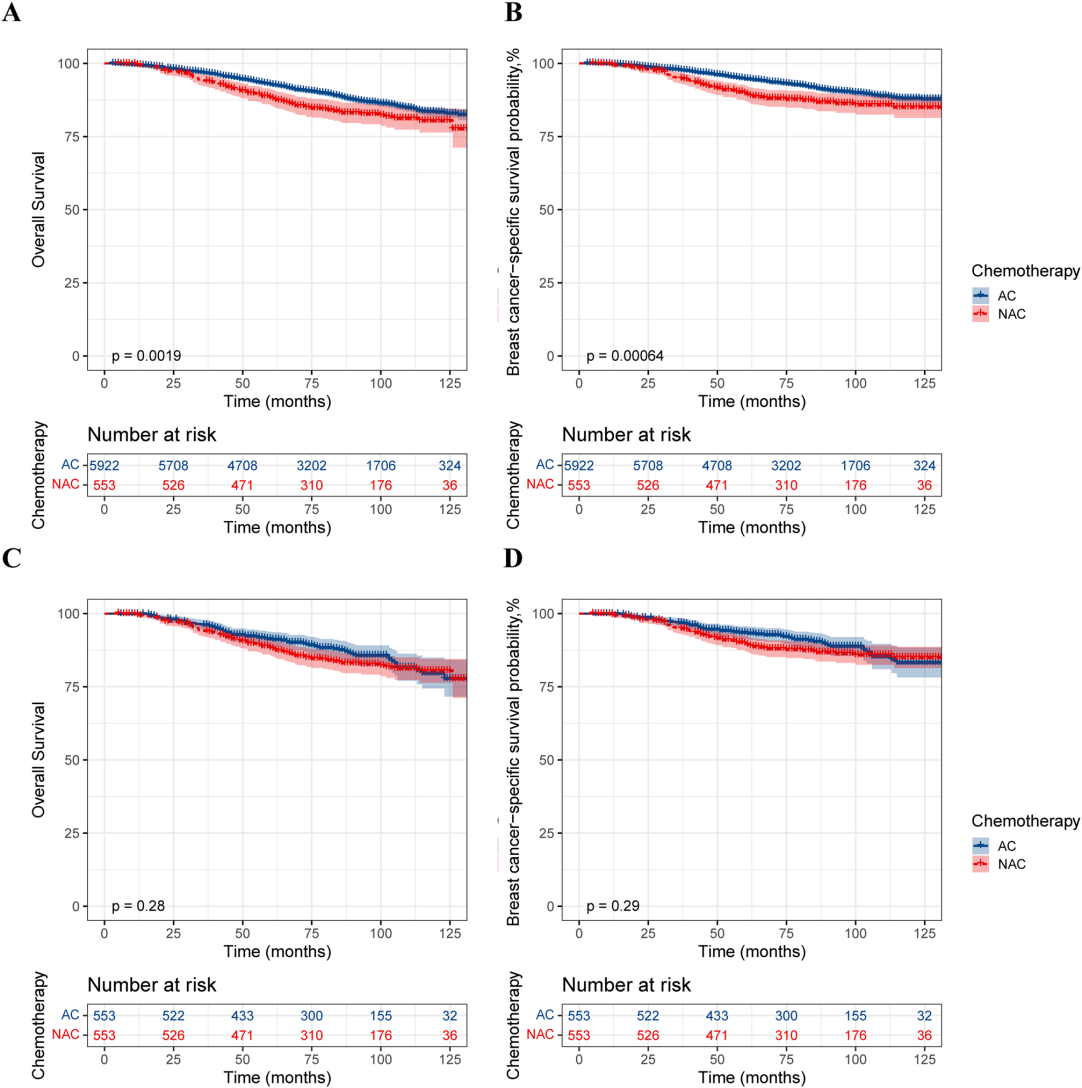
Kaplan‒Meier survival curve of T2N0–1M0 patients. **A** Before PSM, overall survival; **B** Before PSM, breast cancer-specific survival; **C** After PSM, overall survival; **D** After PSM, breast cancer-specific survival

**Fig. 3:**
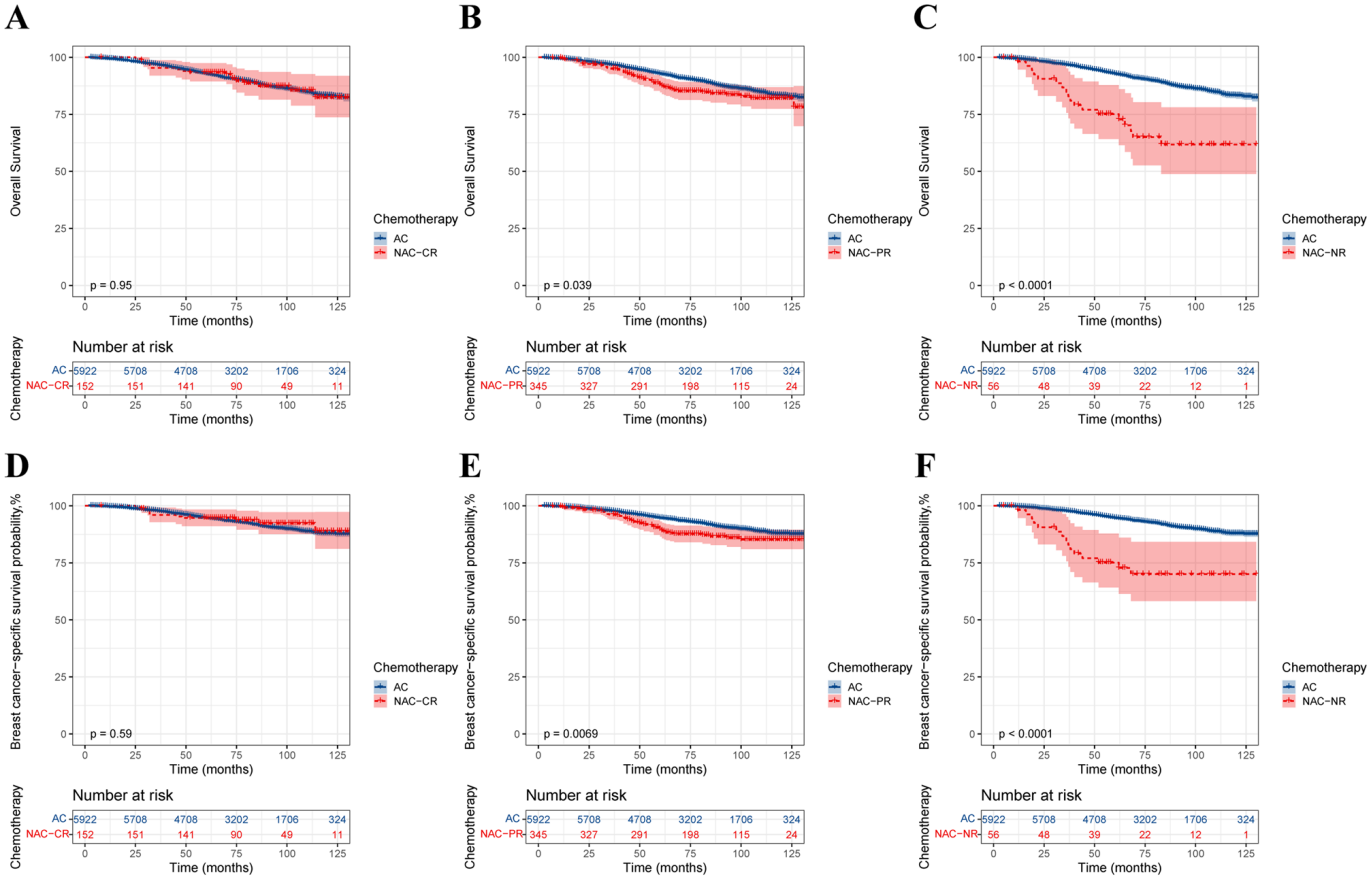
Kaplan‒Meier survival curves for T2N0–1M0 patients stratified by response to NAC. **A** NAC-CR, OS; **B** NAC-PR, OS; **C** NAC-NR, OS; **D** NAC-CR, BCSS; **E** NAC-PR, BCSS; **F** NAC-NR, BCSS

The Kaplan‒Meier method was used to construct survival curves for T2N0–1M0 BC patients. Before PSM, the AC group demonstrated better OS (p = 0.0019) and BCSS (p = 0.00064) than did the NAC group (Fig. 2A and B). However, after PSM, there was no significant difference in OS (p = 0.28) and BCSS (p = 0.29) between the NAC and AC groups (Fig. 2C and D). Stratified analysis of the response to NAC suggested that for complete response (CR) patients, the survival effects of NAC and AC were approximately the same. However, NAC conferred worse survival in the no response (NR) and partial response (PR) populations (Fig. 3). The results of the subsequent Cox proportional hazards regression analysis showed that all the variables analysed were independent prognostic factors in this population. Radiation after BCS is an independent protective factor for OS in this population but is not related to BCSS. For NR and PR patients, NAC was considered to be an independent risk factor for OS [NR, (HR 3.72; 95% CI 2.32–5.95; P < 0.001) PR, (HR 1.39; 95% CI 1.05–1.84; P = 0.023)] and BCSS [NR, (HR 4.51; 95% CI 2.69–7.56; P < 0.001) PR, (HR 1.54; 95% CI 1.13–2.11; P = 0.007)] (Table 2). Overall, NAC followed by BCS did not confer a survival benefit compared with BCS followed by AC in the T2N0-1M0 population. NAC even demonstrated worse survival in the NR and PR populations.

**Table 2:** OS and BCSS in univariate and multivariate analyses

Variable	T2N0-1M0							
	OS				BCSS			
	Univariate		Multivariate		Univariate		Multivariate	
	HR (95%CI)	P	HR (95%CI)	P	HR (95%CI)	P	HR (95%CI)	P
Age								
< = 50	Reference		Reference		Reference		Reference	
> 50	1.42(1.21–1.67)	< 0.001	1.43(1.21–1.68)	< 0.001	1.07(0.89–1.28)	0.466	1.09(0.90–1.30)	0.377
Race								
Black	Reference		Reference		Reference		Reference	
Other^a^	0.43(0.31–0.60)	< 0.001	0.53(0.38–0.75)	< 0.001	0.52(0.35–0.76)	< 0.001	0.63(0.43–0.93)	0.020
White	0.78(0.64–0.96)	0.018	0.91(0.74–1.12)	0.377	0.82(0.64–1.05)	0.115	0.98(0.76–1.26)	0.880
Marital_status								
Married	Reference		Reference		Reference		Reference	
Not-married^b^	1.58(1.37–1.82)	< 0.001	1.50(1.29–1.74)	< 0.001	1.41(1.19–1.68)	< 0.001	1.36(1.14–1.62)	0.001
Histologic_type								
Ductal	Reference		Reference		Reference		Reference	
Ductal and lobular	0.62(0.44–0.88)	0.007	0.68(0.48–0.97)	0.031	0.50(0.32–0.78)	0.002	0.59(0.38–0.93)	0.022
Lobular	0.71(0.52–0.96)	0.028	0.85(0.62–1.16)	0.297	0.57(0.38–0.84)	0.005	0.79(0.53–1.19)	0.259
Grade								
Grade I	Reference		Reference		Reference		Reference	
Grade II	1.31(0.93–1.86)	0.124	1.35(0.95–1.92)	0.091	1.89(1.13–3.16)	0.015	1.92(1.15–3.21)	0.013
Grade III or IV	2.21(1.57–3.10)	< 0.001	2.18(1.55–3.08)	< 0.001	3.91(2.37–6.46)	< 0.001	3.83(2.31–6.35)	< 0.001
Radiation								
No	Reference		Reference		Reference		Reference	
Yes	0.72(0.60–0.87)	< 0.001	0.75(0.62–0.89)	0.002	0.81(0.65–1.01)	0.059	0.83(0.66–1.04)	0.099
Chemotherapy-MERGE								
AC	Reference		Reference		Reference		Reference	
NAC	1.42(1.14–1.77)	0.002	1.40(1.12–1.75)	0.003	1.55(1.20–1.99)	< 0.001	1.47(1.14–1.89)	0.003
Chemotherapy								
AC	Reference		Reference		Reference		Reference	
NAC-CR	0.98(0.62–1.57)	0.950	0.88(0.55–1.40)	0.579	0.85(0.47–1.54)	0.585	0.69(0.38–1.26)	0.225
NAC-NR	3.59(2.25–5.74)	< 0.001	3.72(2.32–5.95)	< 0.001	4.19(2.51–7.01)	< 0.001	4.51(2.69–7.56)	< 0.001
NAC-PR	1.34(1.01–1.78)	0.039	1.39(1.05–1.84)	0.023	1.54(1.12–2.10)	0.007	1.54(1.13–2.11)	0.007

^a^Other American Indian/AK Native, Asian/Pacific Islander

^b^Not-married single (never married), separated, divorced, widowed, unmarried or domestic partner

### Survival analysis of the T2N0M0 and T2N1M0 subgroups

**Fig. 4:**
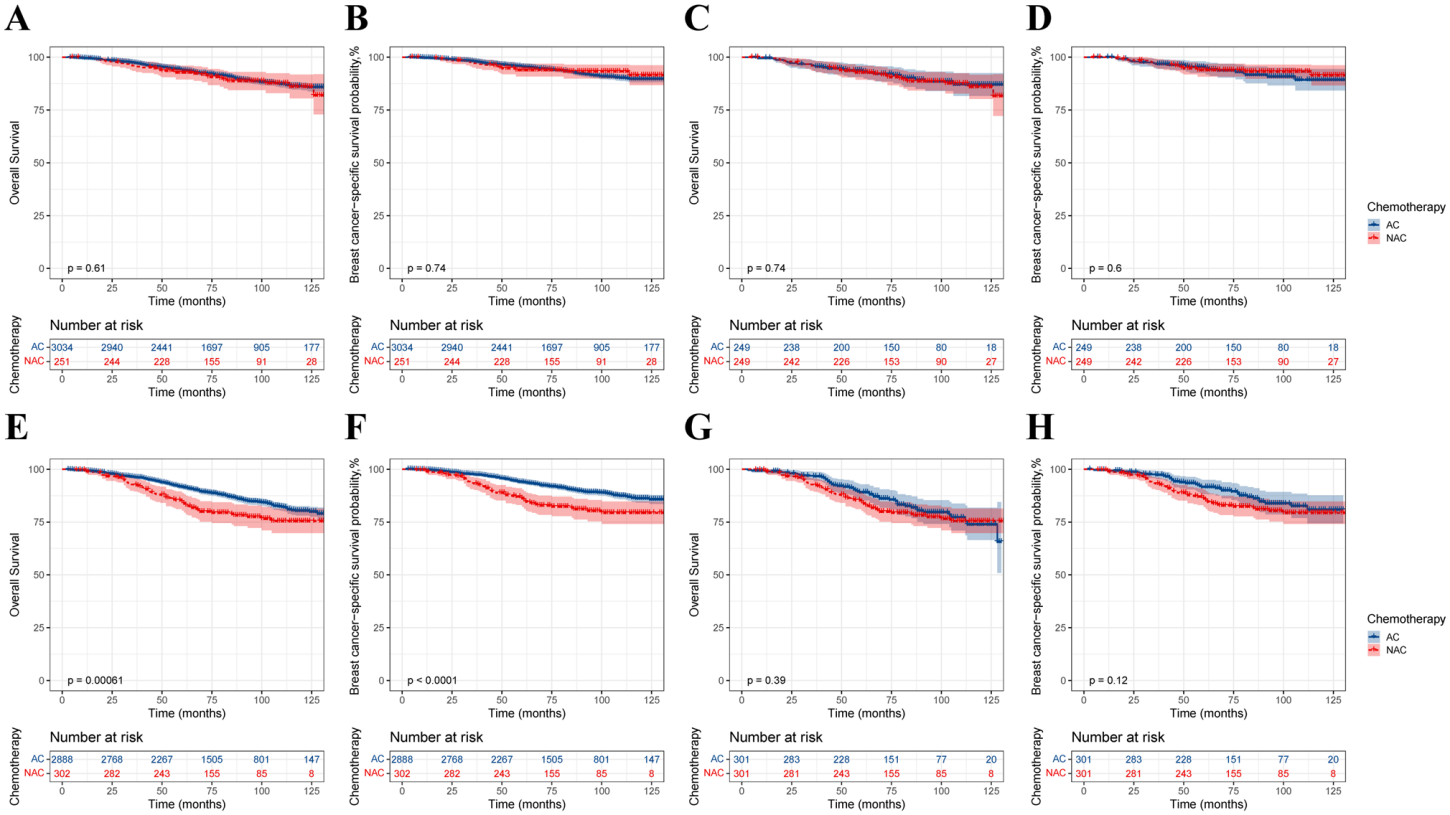
Kaplan‒Meier survival curves for patients with the T2N0M0 subgroup and T2N1M0 subgroup. **A** T2N0M0, before PSM, overall survival; **B** T2N0M0, before PSM, breast cancer-specific survival; **C** T2N0M0, after PSM, overall survival; **D** T2N0M0, after PSM, breast cancer-specific survival; **E** T2N1M0, before PSM, overall survival; **F** T2N1M0, before PSM, breast cancer-specific survival; **G** T2N1M0, after PSM, overall survival; **H** T2N1M0, after PSM, breast cancer-specific survival

**Fig. 5:**
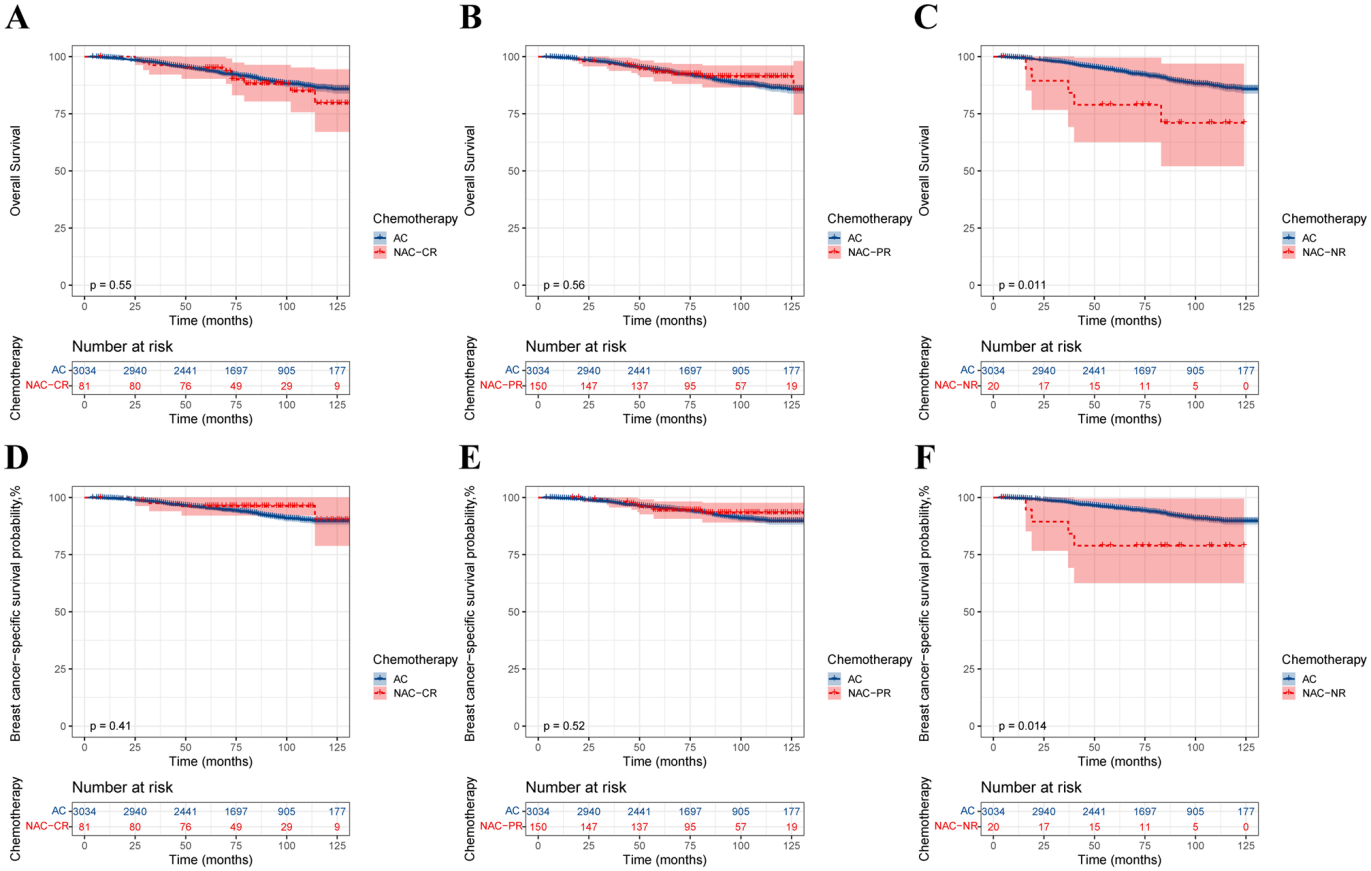
Kaplan‒Meier survival curves for T2N0M0 patients stratified by response to NAC. **A** NAC-CR, OS; **B** NAC-PR, OS; **C** NAC-NR, OS; **D** NAC-CR, BCSS; **E** NAC-PR, BCSS; **F** NAC-NR, BCSS

**Fig. 6:**
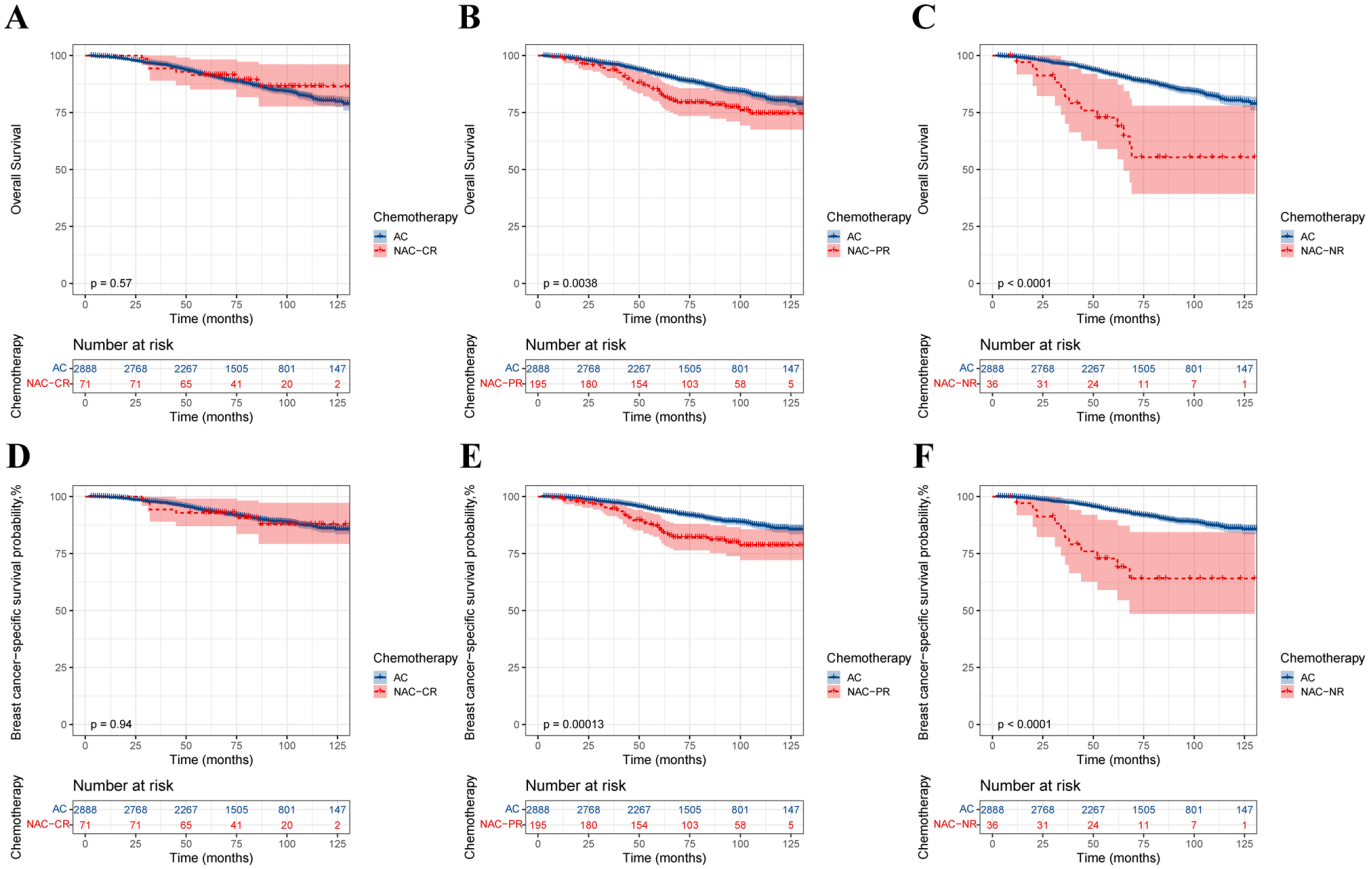
Kaplan‒Meier survival curves for T2N1M0 patients stratified by response to NAC. **A** NAC-CR, OS; **B** NAC-PR, OS; **C** NAC-NR, OS; **D** NAC-CR, BCSS; **E** NAC-PR, BCSS; **F** NAC-NR, BCSS

To further investigate the effect of NAC on survival, the population was divided into T2N0M0 and T2N1M0 subgroups according to N stage, and baseline statistics and PSM were performed for each subgroup (Supplementary Table 1). In the T2N0M0 group, there were no statistically significant differences in OS and BCSS between the AC and NAC groups before and after PSM (Fig. 4A–D). After stratification of patients according to their response to NAC, except for the NR population, the CR and PR populations both showed no difference in survival between the two groups (Fig. 5). In the T2N1M0 subgroup, before PSM, the NAC group had worse OS and BCSS than did the AC group (Fig. 4E–F). However, after PSM, there was no significant difference in OS and BCSS between the AC and NAC groups (Fig. 4G–H). When stratified according to the response to NAC, there was no significant difference in OS and BCSS between the two groups of CR patients. However, both NR and PR patients who received NAC still had worse survival (Fig. 6).

Age was strongly associated with treatment choice and treatment efficacy in the HR-positive/HER2-negative population, so we calculated age-AHR for the two subgroups based on multivariate Cox regression (Table 3). In the T2N0M0 subgroup, NAC did not improve survival compared with the AC group, regardless of menopausal status. However, after stratification, receiving NAC was a risk factor in the < 50 years NR population.

**Table 3:** Age-adjusted hazard ratios of OS and BCSS for patients

			NAC		NAC-CR		NAC-NR		NAC-PR	
			AHR (95% CI)	P	AHR (95% CI)	P	AHR (95% CI)	P	AHR (95% CI)	P
T2N0M0	Age	< = 50								
		OS	0.80(0.38–1.65)	0.541	0.49(0.12–2.00)	0.319	8.02(1.89–34.06)	0.005	0.70(0.25–1.92)	0.487
		BCSS	0.93(0.45–1.95)	0.853	0.56(0.14–2.28)	0.417	11.05(2.56–47.74)	0.001	0.83(0.30–2.28)	0.716
		> 50								
		OS	1.24(0.78–1.96)	0.367	1.42(0.70–2.89)	0.333	2.82(0.90–8.87)	0.076	0.95(0.48–1.85)	0.871
		BCSS	0.83(0.42–1.64)	0.593	0.52(0.13–2.11)	0.360	2.60(0.64–10.57)	0.180	0.80(0.33–1.96)	0.629
T2N1M0	Age	< = 50								
		OS	1.84(1.18–2.89)	0.007	1.14(0.41–3.14)	0.805	3.82(1.64–8.90)	0.002	1.76(1.01–3.04)	0.045
		BCSS	2.12(1.34–3.35)	0.001	1.25(0.45–3.47)	0.668	4.68(1.99–10.97)	< 0.001	2.03(1.17–3.55)	0.012
		> 50								
		OS	1.41(0.99–2.01)	0.054	0.50(0.19–1.35)	0.173	2.92(1.38–6.22)	0.005	1.63(1.08–2.46)	0.020
		BCSS	1.58(1.05–2.38)	0.029	0.53(0.17–1.66)	0.273	3.42(1.40–8.36)	0.007	1.88(1.17–3.03)	0.009

### Nomograms

**Fig. 7:**
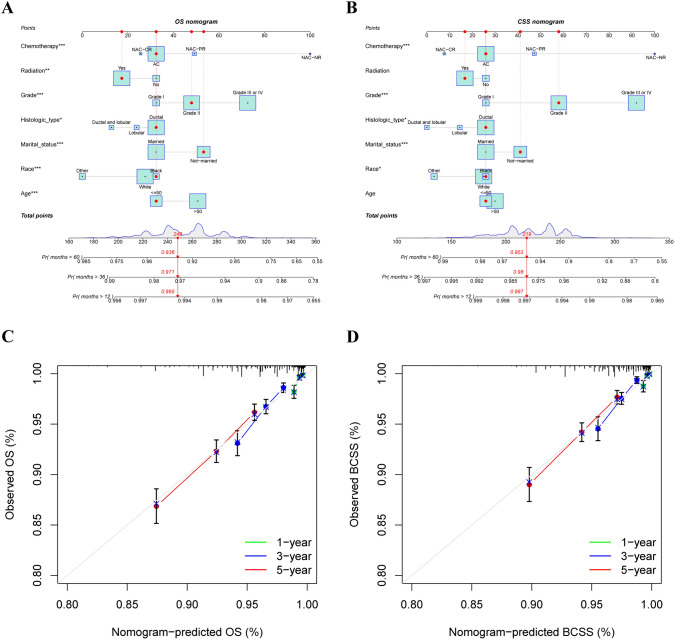
1-, 3-, and 5-year survival probability prediction models. **A** Nomogram model for predicting OS; **B** Nomogram model for predicting BCSS; **C** The calibration curve of the OS nomogram; **D** The calibration curve of the BCSS nomogram

According to the results of the Cox regression analysis, age, race, marital status, histological type, differentiation grade, postoperative radiotherapy and neoadjuvant chemotherapy were determined to be independent prognostic factors. Independent prognostic factors were included in the construction of the nomogram. Different prognostic factor variables were assigned their specific scores, and cumulative scores were compared with linear predictors to yield probabilistic predictions of OS and BCSS at 1, 3, and 5 years (Fig. 7A and B). The c-index of the nomograms for predicting OS and BCSS were 0.646 and 0.662, respectively. Calibration curves further demonstrated the robust predictive performance of the model (Fig. 7C and D).

## Discussion

NAC has the advantages of shrinking the primary tumor, reducing surgical trauma and monitoring the response to chemotherapy, but it has the disadvantages of delaying surgical treatment, inducing chemotherapy resistance and increasing the risk of disease progression (Hönig et al. 2004; Ikeda et al. 2002). Therefore, the use of NAC is controversial for some chemotherapy-insensitive tumor types.

For stage II BC patients, NAC can provide the clinical advantage of tumor downstaging, especially in HER2-positive and TNBC patients (Burstein et al. 2021). According to the 2019 St. Gallen expert consensus, preoperative systemic therapy is preferred for stage II HER2-positive patients and TNBC patients (Burstein et al. 2019). For HR-positive/HER2-negative patients, surgical resection is often the first choice, followed by NAC, neoadjuvant endocrine therapy, and systemic adjuvant therapy based on clinicopathological characteristics (Cantini et al. 2024). The distribution of our included baseline population was the same, with 91.5% of patients choosing BCS combined with AC and only 8.5% choosing NAC combined with BCS. Although the pCR rate of NAC in HR-positive/HER2-negative patients is very low, its clinical response rate can reach 70% (Alba et al. 2012), and evidence supports that NAC can achieve a 60% BCS conversion rate in this population (Torrisi et al. 2021), thus NAC is recommended only for patients who require downsizing for BCS (Torrisi et al. 2021). Our study revealed that for patients with stage T2N0-1M0 disease who could have undergone BCS, preoperative NAC did not confer a significant survival benefit. The survival of patients treated with NAC was comparable to or slightly worse than that of patients treated with AC. Interestingly, no significant difference in survival was observed between CR patients and those who received AC, whereas NR and PR patients demonstrated worse survival than those who received AC. A previous study reported the same efficacy of NAC and AC for patients with stage II BC (Fisher et al. 2023), which may be the reason why the survival of CR patients is roughly the same as that of patients who achieved AC. However, PR and NR patients are not sensitive to chemotherapy drugs. For patients who can undergo direct surgery, NAC actually prolongs the ineffective treatment time and delays the effective surgical treatment. During this period of NAC treatment, it may not only induce clonal expansion of drug-resistant cells but also promote disease progression and ultimately lead to worse survival (Hönig et al. 2004). In conclusion, NAC does not result in better survival than AC in patients with T2N0-1M0 HR-positive/HER2-negative BC for whom BCS is feasible; therefore, NAC is not recommended for this population.

Several studies have suggested that the efficacy of NAC is closely related to age (Boughey et al. 2022; Loibl et al. 2015; Verdial et al. 2022). Although the survival of younger breast cancer patients is worse, the pCR rate of NAC in the younger HR-positive/HER2-negative population was significantly greater than that in older patients (Loibl et al. 2015). Moreover, younger women who underwent NAC had a greater nodal pCR rate and greater axillary downstaging (Boughey et al. 2022; Verdial et al. 2022). However, our findings suggest that NAC is not recommended for chemotherapy-insensitive patients of all ages. This result could also be attributed to the apparent effect of NAC in delaying effective treatment in a chemotherapy-insensitive population. In the era of precision medicine, it is essential to identify biomarkers or predictive models that can predict the survival of early HR-positive/HER2-negative patients (Cantini et al. 2024). The nomogram model robustly predicted the 1-, 3-, and 5-year OS and BCSS of T2N0-1M0 HR-positive/HER2-negative patients who underwent BCS. Cox analysis revealed that postoperative radiotherapy was an independent protective factor for OS in patients with T2N0-1M0 HR-positive/HER2-negative BC, but it was not associated with BCSS. HR-positive/HER2-negative BC generally has a more favourable outcome after radiotherapy than other types of breast cancer (He et al. 2019; Hung et al. 2023). This may be related to the increase in radiosensitivity caused by the interaction between estrogen receptors and androgen receptors (Michmerhuizen et al. 2022). In addition, radiotherapy after BCS can also reduce the possibility of tumor metastasis (Hung et al. 2023) and improve the effect of immunotherapy (Yu et al. 2019). These factors may account for the overall survival advantage of radiotherapy after BCS in our study population.

We must acknowledge and accept the limitations of our study. First, we cannot avoid the bias caused by the retrospective nature of the study, but we balanced group differences through PSM to enhance comparability and minimize the impact of bias. Second, information on Ki67 was missing in the SEER database, so we could not perform an analysis of this critical factor. Finally, more detailed treatment information is not available in the SEER database, and we plan to obtain more comprehensive data to validate our results in future clinical work.

In general, NAC before BCS is not necessary for T2N0-1M0 HR-positive/HER2-negative BC patients.

## Supplementary Information

Below is the link to the electronic supplementary material.

Supplementary file1 (XLSX 13 KB)

## Data Availability

The datasets analysed during the current study are available in the Surveillance, Epidemiology, and End Results (SEER) Program (www.seer.cancer.gov). SEER*Stat Database: Incidence—SEER Research Data, 17 Registries, Nov 2022 Sub (2000–2020).
